# Prostatic Urethral Lift (PUL) for obstructive median lobes: 12 month results of the MedLift Study

**DOI:** 10.1038/s41391-018-0118-x

**Published:** 2018-12-12

**Authors:** Daniel Rukstalis, Douglas Grier, Sean P. Stroup, Ronald Tutrone, Euclid deSouza, Sheldon Freedman, Richard David, Jed Kamientsky, Gregg Eure

**Affiliations:** 1 Wake Forest University Health Sciences Medical Center Blvd, Winston-Salem, NC 27157 USA; 2 Sound Urological Associates, 21822 76th Avenue West Edmonds, Edmonds, WA 98026 USA; 3 Department of Urology, Naval Medical Center San Diego, 34800 Bob Wilson Drive San Diego, San Diego, CA 92134 USA; 4 Chesapeake Urology Research Associates, 6535 N, Charles St., Suite 625, Towson, MD 21204 USA; 5 Adult & Pediatric Urology, PC 10707 Pacific Street Suite 101, Omaha, NE 68114 USA; 6 Sheldon J. Freedman, M.D., LTD 653 N Town Center Drive, Suite 308, Las Vegas, NV 89144 USA; 7 Skyline Urology, 5522 Sepulveda Blvd Sherman Oaks, Oaks, CA 91411 USA; 8 Manhattan Medical Research Practice, PLLC 215 Lexington Avenue 21th Floor, New York, 10016 USA; 9 Urology of Virginia, PLLC 225 Clearfield Ave, Virginia Beach, VA 23462 USA

**Keywords:** Outcomes research, Urogenital diseases, Prostatic diseases

## Abstract

Evidence indicating Prostatic Urethral Lift (PUL) delivers significant improvement in symptomatic BPH with low morbidity is based on subjects with lateral lobe (LL) enlargement only. MedLift was an FDA IDE extension of the L.I.F.T. randomized study designed to examine safety and efficacy of PUL for treatment of obstructive middle lobes (OML). Inclusion criteria for this non-randomized cohort were identical to the L.I.F.T. randomized study, except for requiring an OML: ≥ 50 years of age, IPSS ≥ 13, and Qmax ≤ 12 ml/s. Primary endpoint analysis quantified improvement in IPSS over baseline and rate of post-procedure serious complications. Quantification of symptom relief, quality of life, flow rate, and sexual function occurred through 12 months. Outcomes were compared to historical L.I.F.T LL results and were combined to demonstrate the full effectiveness of PUL. Of the 71 screened subjects, 45 were enrolled. At 1, 3, 6, and 12 months, mean IPSS improved from baseline at least 13.5 points (*p* < 0.0001). Quality of life and BPHII were similarly improved (>60% and >70%, respectively at 3, 6, and 12 months, *p* < 0.0001). Mean Qmax improvement ranged from 90 to 129% (*p* < 0.0001). At 1 month, 86% (CI 73–94%) reported ≥70 on the Quality of Recovery scale, 80% (CI 66–89%) reported being “much” or “very much better,” and 89% (CI 76–95%) would recommend the procedure. Compared to LL subjects, OML subjects’ symptoms improved at least as much at every time point (OML range 13.5–15.9, LL range 9.9–11.1, *p* ≤ 0.01). On combining OML with LL data, >70% (range CI 63–81%) of subjects demonstrated ≥ 8 point improvement in IPSS through 12 months. Analysis of the combined dataset indicates ≥ 40% (CI 30–51%) of sexually active men improved the minimal clinically important difference in erectile function through 12 months. Prostates, including those with middle lobe obstruction, can be treated with the PUL procedure safely and effectively.

## Introduction

The Prostatic Urethral Lift (PUL) procedure is a minimally invasive option for lower urinary tract symptoms (LUTS) in patients with bladder outlet obstruction (BOO) that provides significant and rapid symptom improvement with low morbidity. Medical therapy is often a first-line treatment but is associated with a risk of side-effects such as 4–15% asthenia, 5–15% dizziness, 5–12% headaches, and 1–10% sexual dysfunction [1]. A large study of 13,474 BPH patients from a U.S. medical claims database found that 61.2% of patients were not adherent to their BPH medications within the first 6 months of alpha blocker use and 66.1% discontinued within the first year [2]. Surgical approaches such as transurethral resection of the prostate (TURP) and photoselective vaporization of the prostate (PVP) provide excellent symptom relief (14.0–14.9 point International Prostate Symptom Score or IPSS improvements at 1 year) but come with a risk of adverse events such as 0–8% need for blood transfusion, 2–7% rate of urethral stricture, 7–10% risk of erectile dysfunction, and 42–65% rate of ejaculatory dysfunction [1, 3, 4]. Intraprostatic steam injection uses steam to ablate the prostate tissue and delivers effective relief (11.7 point IPSS improvement at 1 year) but comes with unwanted problems including a 3–6% rate of ejaculatory dysfunction, 7–17% rate of urinary tract infection, 90–100% post-operative catheterization, and 14% prolonged catheterization [5–7]. In contrast, the PUL procedure has been shown to be a minimally invasive option that provides rapid, significant relief (IPSS improvement 10.8 points at 1 year) for selected subjects with lateral lobe (LL) obstruction [8–13].

Obstruction due to middle lobe enlargement is less common than LL obstruction. In a study of 157 consecutive male patients age ≥ 50 years presenting with LUTS and IPSS > 7 to a urology center, 70.1% had LL enlargement while 21.6% had middle lobe enlargement as measured by transabdominal ultrasound [14]. In the PUL L.I.F.T. study, 5.3% of those subjects assessed for randomization were excluded for an obstructive median or middle lobe (OML) [13].

Quantifying OML can be a challenge; there are no standardized criteria for measuring the size or degree of obstruction. This could be due in part to the fact that there is wide variation in the severity and morphology of middle lobe enlargement [15]. One measurement approach that has been shown to correlate well with BOO is intravesical prostatic protrusion (IPP), an ultrasonic measurement of prostatic protrusion into the bladder [16–19]. IPP is commonly measured as the vertical distance from the tip of the protruding prostate to the base of the bladder and its severity is often graded [16–18, 20]. A prospective study of 200 men age ≥ 50 years presenting with LUTS found that IPP correlated well with BOO (positive predictive value 94%, negative 79%) and also with the severity of obstruction as defined by a higher BOO index (*p* < 0.001). Almost all patients with high grade IPP had significant obstruction [16].

Treating OML can be a challenge. Middle lobe enlargement and the related IPP are associated with a higher risk of urinary retention and a higher failure rate of medical therapy for LUTS due to BPH [15, 19]. There is also a risk of greater surgical difficulty with this anatomy, particularly when the IPP is severe or there is a high bladder neck [21].

The mechanical approach of retracting enlarged prostatic lobes using small UroLift® implants has been well-studied in men with LL enlargement only [8–13]. The L.I.F.T. study showed that PUL is safe and delivers rapid, significant relief by 2 weeks that is durable to 5 years [13]. Adverse events were mild-moderate and typically resolved by 2–4 weeks [13]. Sexual function was stable over 5 years with no de novo, sustained erectile, or ejaculatory dysfunction [13]. The MedLift study was undertaken as an extension of the L.I.F.T. clinical trial to determine the safety and effectiveness of PUL for OML subjects and to see how the results compare with LL subjects. As a cohort extension study, the enrollment criteria were identical to L.I.F.T. except for requiring the presence of OML. The 12 month follow-up results of this MedLift study are presented herein.

## Materials and methods

### Protocol

A prospective, non-randomized study of the safety and effectiveness of the PUL procedure in subjects with OML was performed in 9 centers across the United States. Enrollment criteria included age ≥ 50 years, IPSS ≥ 13, peak flow rate (Qmax) ≤ 12 mL/s with a 125 mL voided volume and 30–80 cc intraurethral prostatic volume as measured by transrectal ultrasound. Prostates with a variety of middle and median lobe characteristics were included, including morphology traditionally described as ball valve, high bladder neck, median bar, and hypertrophied central zone. OML was defined as excessive posterior tissue that during the pre-treatment cystoscopy the operator thought would preclude a normal LL procedure; prostates with a variety of middle and median lobe character were included, including centrally and circumferentially, symmetrically and asymmetrically elevated tissue. In the opinion of the investigator, the middle or median lobe appeared obstructive and would have contraindicated a purely LL PUL. In this manuscript, we use the terms median and middle lobe interchangeably. Excluded from the study were men who had undergone prior surgical intervention for BPH, current urinary retention, active urinary tract infection, and other potentially confounding conditions. Subjects were required to undergo a washout of 2 weeks for alpha blocker, 3 months for 5 alpha-reductase inhibitor, and 3 days for anticoagulants prior to treatment. In accordance with the Declaration of Helsinki and federal regulations, the study was performed with approval from the institutional review boards and all men gave written informed consent (Clinicaltrials.gov: NCT02625545).

### Procedure

**Fig. 1:**
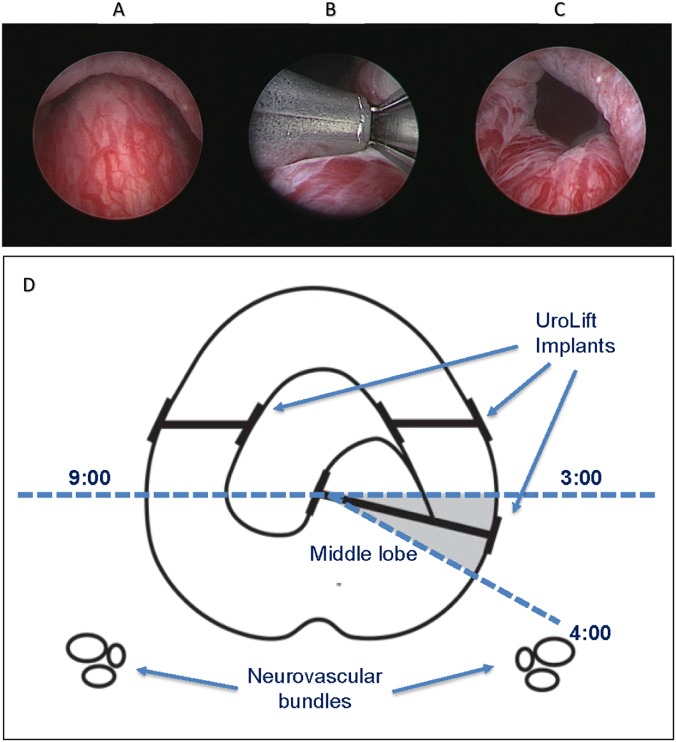
Middle lobe deployment of UroLift system implants. **a** after addressing the lateral lobes, obstructive middle lobe visualized on cystoscopy, **b** UroLift implant is deployed in mostly lateral and slightly posterior direction to secure the middle lobe tissue to the side of the prostatic urethra, **c** bladder neck opening is achieved. **d** It is important to deploy the implant away from the neurovascular bundles, so operators should maintain deployment trajectory anterior to the 4 and 8 o’clock position when viewing the transverse plane of the urethra as a clock face. Photos courtsey of Dr. Gregg Eure.

The PUL procedure involves small permanent metallic implants that are placed under cystoscopic guidance to reduce urethral obstruction by creating an anterior channel through the prostatic fossa. The implant is comprised of a monofilament suture with a metallic capsular tab on one end and a metallic urethral end-piece on the other. The implant is deployed through a delivery device (UroLift® System, NeoTract-Teleflex, Pleasanton, CA) that houses a 2.9 mm telescope and is inserted into the body with the assistance of a 20 F sheath. Prior to deployment, the physician may conduct a cystoscopy to select the target implant locations. For LL deployments, the system is angled laterally (20–30 degrees) usually at the 10 and 2 o’clock position to compress the anterior third of the obstructive lobe. For middle lobe deployments, tissue that protrudes intravesically may be pulled into the prostatic fossa and affixed to either side of the urethra dependent on the individual’s prostate anatomy (Fig. 1). It is important to note that, with large IPP, not all intravesical tissue needs to be retracted when creating the channel at the bladder neck. Additional tissue may remain intravesical, albeit not obstructing the prostatic fossa. During implant deployment, the delivery device advances a 19 gauge needle through the lobe. As the needle is withdrawn, the capsular tab of the implant engages the prostatic capsule. The monofilament is then tensioned, cut to the width of the compressed lobe, and secured in place by the urethral end-piece. Thus, the length of the suture is dependent on the deployment location and is customized to the individual’s prostate anatomy.

### Study assessments

The primary objective was to determine the effectiveness and safety of PUL for treating subjects with OML. The primary endpoint was to demonstrate at 6 months that the mean percent improvement in IPSS over baseline for PUL was > 30%. The study was powered to have 95% probability of establishing the true percent improvement in IPSS score from baseline to 6 months was greater than 25%, with 95% confidence. The minimum required number of evaluable subjects was determined to be 35, assuming the mean % improvement in IPSS was 43.5% with a standard deviation of 31.5%. In addition, the primary safety endpoint was to demonstrate that the composite observed rate of post-procedure device related serious complications was ≤ 15% at 3 months.

Subjects were followed for 1 year and assessed on symptom response (IPSS), quality of life (QoL and BPH Impact Index, BPHII), Qmax, sexual function (International Index of Erectile Function, IIEF, and Male Sexual Health Questionnaire for Ejaculatory Dysfunction, MSHQ-EjD), and adverse events. Protocol required cystoscopy at 6 months ensured that implants would be assessed for the presence of encrustation. An independent clinical events committee adjudicated all adverse events. An independent central reviewer over-read all uroflow waveforms, calculating Qmax using the 2-second rule.

**Fig. 2:**
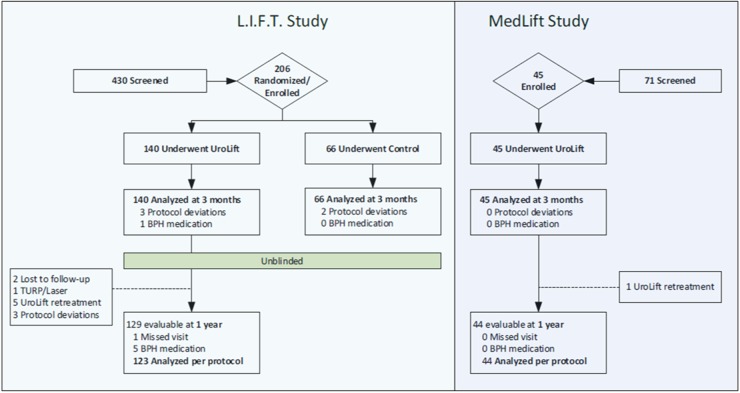
CONSORT diagram of the L.I.F.T. study and MedLift study

Analyses were conducted on an Intent to Treat (ITT) and Per Protocol (PP) basis. One subject was found to have pre-existing conditions including kidney stones and significant cardiac disease that could impact study results and was removed from the PP analysis. The bootstrap method was used to calculate the lower limit of the one-sided 95% confidence interval for the percent improvement in IPSS to test the primary study hypothesis. Paired *t*-tests were used to calculate *p*-values for each follow-up interval compared to baseline. Historical cohort comparison included all L.I.F.T. participants who were still being followed per protocol (Fig. 2). IPP group analysis was performed to compare outcomes across the three IPP categories [Group 1 ( < 5 mm), Group 2 (5–10 mm), and Group 3 ( > 10 mm)] with the Jonckheere–Terpstra test.

## Results

Of the 71 men who were screened for eligibility, 45 were enrolled between March 2016 and January 2017. Baseline characteristics of the OML subjects were similar to the characteristics of the L.I.F.T. active and control arms except the OML cohort like the control arm was younger and more symptomatic per IPSS than the LL cohort (Table 1). Average intraurethral prostate volume was 44 ± 11cc (range 30–68) and average prostate volume including IPP was 53 ± 14cc (range 31–88). All (45 of 45; 100%) procedures initiated were successfully completed. Of the 45 subjects, 23 (51%) received general anesthesia, 16 (36%) received intravenous (IV) sedation only, and 6 (13%) received topical/local anesthesia along with IV sedation. An average of 6.3 implants were used per subject, of which 1.3 implants on average were needed to treat the middle lobe. Average length of stay after procedure was 2.4 h (median 1.8, SD 2.7) with only one subject staying overnight (18.5 h stay).

**Table 1:** Baseline characteristics of obstructive middle lobe (OML, MedLift study), lateral lobe only (LL, L.I.F.T. study), and sham (control, L.I.F.T. study) cohorts

Mean (SD), Median	OML (MedLift)	LL only (L.I.F.T. active)	*p*-value (LL to OML)	Sham (L.I.F.T. control)	*p*-value (Sham to OML)
Age	64 (7.0), 63.0	67 (8.6), 67	0.03	65 (8.0), 64	0.7
Prostate Volume (cc)^a^	44.2 (11.2), 41.3	44.5 (12.5), 42.4	0.9	40.9 (10.8), 38.0	0.1
IPSS	24.2 (4.9), 23.0	22.2 (5.4), 22.0	0.04	24.4 (5.8), 26.0	0.8
MSHQ-EjD function	9.4 (3.1), 10.0	8.7 (3.2), 9.0	0.3	8.8 (3.2), 9.0	0.4
IIEF-5	15.1 (9.0), 19.0	13.0 (8.4), 14.0	0.2	13.5 (8.5), 14.5	0.3
Qmax (mL/sec)	7.2 (2.9), 7.0	7.8 (2.4), 8.0	0.1	7.9 (2.4), 8.0	0.2
PVR	107.3 (79.9), 86.0	85.5 (69.2), 72.0	0.08	87.7 (72.4), 73.5	0.2
Implants per subject	6.3 (1.6), 6.0	5.1 (2.2), 5.0	0.0005	NA	NA
Implants per middle lobe	1.3 (0.8), 1.0	NA	NA	NA	NA

^a^For the MedLift study, the prostate volume did not include the intravesical prostatic protrusion volume

A catheter was placed post-operatively without a voiding trial in 29/45 subjects (64.4%). An additional 7 subjects (15.6%) failed a voiding trial and required a catheter prior to discharge. Mean catheter duration was 1.2 days averaged over the total cohort. Peri-operative adverse events were typically mild to moderate and transient, with the most frequent being hematuria and dysuria. Over the one-year course of the study, few related adverse events occurred after the first month.

Both the effectiveness and safety primary endpoints were met. The mean improvement in IPSS at 6 months was 57.7%, with mean IPSS improvement maintained through 12 months at 55.1%. The observed rate of post-procedure device related serious complications was 0%, thereby achieving the primary safety composite endpoint. There was no significant difference in any efficacy measure between PP and ITT analyses.

Mean IPSS improvement at 1, 3, 6, and 12 months was at least 13.5 points and significantly better than baseline at every time point (Table 2, *p* < 0.0001). QoL and BPHII were similarly improved (>60% and >70%, respectively at 3, 6, and 12 months). Mean Qmax improvement ranged from 90–130% throughout follow up. At 1 month, 65% subjects reported >80 on the Quality of Recovery scale, 95% reported feeling ‘better’ with 80% feeling ‘much’ or ‘very much better,’ and 89% would recommend the procedure. By 3 months, 93% would recommend the procedure.

**Table 2:** Relevant outcomes for OML (MedLift) subjects and combined data (OML with LL data) from the pivotal L.I.F.T

Test/ Procedure		1 Month		3 Months		6 Months		12 Months	
		OML	Combined	OML	Combined	OML	Combined	OML	Combined
IPSS	N (paired)	45	180	45	181	45	178	44	167
	Baseline	24.2 ± 4.9	22.7 ± 5.4	24.2 ± 4.9	22.8 ± 5.4	24.2 ± 4.9	22.7 ± 5.4	24.1 ± 5.0	22.7 ± 5.5
	Follow-up	9.8 ± 5.7	11.7 ± 6.7	8.3 ± 5.1	10.4 ± 7.2	10.0 ± 6.4	10.9 ± 7.1	10.6 ± 7.0	11.3 ± 7.2
	Change	−14.4 ± 6.7	−11.1 ± 7.2	−15.9 ± 6.8	−12.3 ± 7.8	−14.2 ± 7.6	−11.8 ± 7.7	−13.5 ± 7.7	−11.4 ± 7.7
	% Change	−59.0% ± 23.9%	−47.8% ± 27.8%	−64.9% ± 21.7%	−53.5% ± 30.0%	−57.7% ± 26.7%	−51.2% ± 30.3%	55.1% ± 28.1%	−49.4% ± 30.5%
	*p*-value	<0.0001	<0.00001	<0.0001	<0.00001	<0.0001	<0.00001	<0.0001	<0.00001
	*p*-value comparison*	0.0004		0.0003		0.01		0.03	
QOL	N (paired)	45	180	45	181	45	178	44	167
	Baseline	4.9 ± 0.8	4.7 ± 1.0	4.9 ± 0.8	4.7 ± 1.0	4.9 ± 0.8	4.7 ± 1.0	4.9 ± 0.8	4.7 ± 1.0
	Follow up	1.8 ± 1.2	2.4 ± 1.6	1.6 ± 1.3	2.2 ± 1.7	1.9 ± 1.4	2.1 ± 1.6	1.9 ± 1.3	2.2 ± 1.5
	Change	−3.1 ± 1.5	−2.3 ± 1.7	−3.3 ± 1.5	−2.5 ± 1.8	−3.0 ± 1.6	−2.6 ± 1.7	−3.0 ± 1.5	−2.5 ± 1.6
	% Change	−61.6% ± 25.6%	−47.0% ± 36.2%	−66.9% ± 26.8%	−51.7% ± 36.7%	−59.9% ± 29.5%	−54.2% ± 33.7%	−61.1% ± 27.7%	−53.4% ± 33.2%
	*p*-value	<0.0001	<0.00001	<0.0001	<0.00001	<0.0001	<0.00001	<0.0001	<0.00001
	*p*-value comparison*	0.0004		0.0003		0.06		0.01	
BPHII	N (paired)	45	180	45	181	45	178	44	167
	Baseline	7.7 ± 2.8	7.1 ± 2.8	7.7 ± 2.8	7.1 ± 2.8	7.7 ± 2.8	7.1 ± 2.8	7.7 ± 2.8	7.0 ± 2.8
	Follow up	3.7 ± 2.5	3.9 ± 2.9	1.8 ± 1.9	2.6 ± 2.8	1.7 ± 1.6	2.4 ± 2.6	2.1 ± 2.5	2.6 ± 2.8
	Change	−4.0 ± 3.4	−3.1 ± 3.5	−5.9 ± 3.4	−4.5 ± 3.4	−6.0 ± 3.2	−4.7 ± 3.3	−5.6 ± 3.5	−4.4 ± 3.4
	% Change	−44.1% ± 48.2%	−35.6% ± 71.4%	−72.9% ± 33.9%	−60.2% ± 44.3%	−75.0% ± 23.6%	−63.9% ± 36.9%	−70.4% ± 37.7%	−60.8% ± 44.0%
	*p*-value	<0.0001	<0.00001	<0.0001	<0.00001	<0.0001	<0.00001	<0.0001	<0.00001
	*p*-value comparison*	0.05		0.0007		0.0017		0.007	
Q_MAX_	N (paired)	37	37	40	162	41	41	37	140
	Baseline	7.2 ± 2.7	7.2 ± 2.7	7.2 ± 2.6	7.8 ± 2.5	7.1 ± 2.6	7.1 ± 2.6	7.1 ± 2.7	7.8 ± 2.5
	Follow up	15.0 ± 7.3	15 ± 7.33	14.6 ± 6.2	12.9 ± 5.6	12.3 ± 5.1	12.3 ± 5.1	13.5 ± 7.6	12.5 ± 6.0
	Change	7.8 ± 6.9	7.8 ± 6.9	7.4 ± 6.2	5.0 ± 5.6	5.2 ± 4.5	5.2 ± 4.5	6.4 ± 7.4	4.7 ± 5.8
	% Change	128.9% ± 118.7%	129% ± 119%	127.3% ± 134.4%	80.0% ± 101%	89.8% ± 99.3%	89.8% ± 99.3%	108.4% ± 133.3%	71.7% ± 98.6%
	*p*-value	<0.0001	<0.00001	<0.0001	<0.00001	<0.0001	<0.00001	<0.0001	<0.00001
	*p*-value comparison*			0.002				0.08	
MSHQ-EjD Function	N (paired)	35	123	36	127	38	132	38	125
	Baseline	9.2 ± 3.1	9 ± 3.1	9.4 ± 3.1	8.9 ± 3.1	9.4 ± 3.1	9.0 ± 3.2	9.4 ± 3.1	8.9 ± 3.2
	Follow up	11.4 ± 3.1	11.3 ± 3.2	11.3 ± 3.4	11.1 ± 3.2	11.2 ± 3.1	10.7 ± 3.2	11.4 ± 2.8	10.6 ± 3.1
	Change	2.2 ± 2.5	2.3 ± 2.9	1.9 ± 2.9	2.2 ± 2.7	1.8 ± 2.8	1.8 ± 2.8	2.0 ± 2.8	1.7 ± 2.7
	% Change	36.2% ± 47.2%	36.2% ± 52.8%	26.3% ± 43.0%	33.2% ± 49.0%	26.6% ± 45.1%	33.2% ± 81.4%	38.8% ± 74.2%	30.9% ± 57.4%
	*p*-value	<0.0001	<0.00001	0.0008	<0.00001	0.0009	<0.00001	0.0026	<0.00001
	*p*-value comparison*	0.9		0.4		1.0		0.4	
MSHQ-EjD Bother	N (paired)	35	123	36	127	38	132	38	125
	Baseline	1.6 ± 1.8	2.0 ± 1.7	1.6 ± 1.7	2.0 ± 1.7	1.6 ± 1.7	2.0 ± 1.7	1.6 ± 1.7	2 ± 1.7
	Follow up	1.1 ± 1.2	1.2 ± 1.4	0.7 ± 1.2	1.0 ± 1.3	0.6 ± 1.2	1.1 ± 1.3	0.9 ± 1.3	1.3 ± 1.4
	Change	−0.5 ± 1.6	−0.8 ± 1.6	−0.9 ± 1.7	−1.0 ± 1.5	−1.0 ± 1.7	−1.0 ± 1.6	−0.6 ± 1.8	−0.7 ± 1.6
	% Change	−32.9% ± 56.3%	−33.2% ± 63.2%	−59.8% ± 54.8%	−50.6% ± 57.0%	−71.2% ± 46.1%	−47.7% ± 55.0%	−51.5% ± 61.7%	−34.4% ± 66.2%
	*p*-value	0.02	<0.00001	<0.0001	<0.00001	<0.0001	<0.00001	0.0008	<0.00001
	*p*-value comparison*	0.3		0.6		0.8		0.7	
IIEF-EF	N (paired)	35	123	36	127	38	132	37	124
	Baseline	22.1 ± 8.4	20.8 ± 8.2	22.4 ± 8.1	20.8 ± 8.1	22.5 ± 7.9	21.0 ± 8.0	21.8 ± 8.6	20.5 ± 8.4
	Follow up	23.4 ± 8.9	22.0 ± 8.7	23.5 ± 8.7	22.2 ± 8.6	21.9 ± 9.5	21.6 ± 8.9	22.6 ± 9.6	21.2 ± 9.1
	Change	1.3 ± 3.6	1.2 ± 5.8	1.1 ± 5.3	1.4 ± 5.4	−0.6 ± 7.0	0.6 ± 5.7	0.8 ± 4.4	0.7 ± 5.7
	% Change	7.0% ± 23.2%	12.5% ± 43.4%	7.9% ± 31.3%	11.6% ± 35.7%	−0.2% ± 32.1%	6.95% ± 36.7%	5.4% ± 28.5%	11.7% ± 72.3%
	*p*-value	0.08	0.02	0.1	0.004	1.0	0.2	0.3	0.2
	*p*-value comparison*	0.9		0.7		0.1		0.9	
SHIM	N (paired)	35	123	36	127	38	132	38	125
	Baseline	17.3 ± 7.6	16.6 ± 7.3	17.5 ± 7.5	16.5 ± 7.2	17.6 ± 7.4	16.7 ± 7.1	17.2 ± 7.8	16.4 ± 7.3
	Follow up	18.6 ± 8.1	17.6 ± 7.8	18.7 ± 7.8	17.8 ± 7.6	17.3 ± 8.4	17.3 ± 7.8	18.4 ± 8.3	17.2 ± 7.9
	Change	1.3 ± 3.6	1.07 ± 5.0	1.3 ± 4.5	1.3 ± 4.6	−0.4 ± 6.1	0.7 ± 4.9	1.2 ± 4.3	0.9 ± 4.9
	% Change	12.6% ± 41.5%	15.5% ± 56.9%	12.4% ± 36.2%	13.9% ± 40.4%	2.2% ± 42.9%	8.87% ± 43.3%	12.3% ± 35.1%	16.6% ± 88.6%
	*p*-value	0.08	0.02	0.05	0.002	0.8	0.1	0.04	0.05
	*p*-value comparison*	0.7		1.0		0.1		0.6	

Study and the MedLift extension study

*p-value comparison represents MedLift to LIFT active cohort only comparison

IPP subgroup analysis of OML subjects indicated that IPP severity did not show significance as a baseline predictor of symptom response (*p* = 0.7). Group 1 with IPP ≤ 5 mm (range 0.8–5.0 mm) demonstrated 12.3 (SD 5.5) improvement from baseline; group 2 with IPP 5–10 mm (range 5.8–9.5 mm) demonstrated 14.4 (SD 6.9) improvement from baseline and group 3 with IPP > 10 mm (range 10.6–36.6 mm) demonstrated 12.9 (SD 8.8) improvement from baseline. Qmax was also not correlated with IPP severity (*p* = 0.4).

Sexual function was preserved with no PUL subjects reporting de novo sustained ejaculatory or erectile dysfunction. There was no significant degradation in mean erectile function (IIEF-5) or ejaculatory function (MSHQ-EjD Function) over the course of follow up (Table 2). Bother due to ejaculatory function improved rapidly and remained modestly improved at 1 year, *p* = 0.001.

At one-year follow up, no subject had been lost to follow up or exited the study. No subject required BPH LUTS medications for return of symptoms. Surgical retreatment for failure to cure occurred in 1 subject (2%) who received additional PUL implants at 9 months with no adverse effect from the presence of implants. No implant was observed to have developed encrustation or stone formation throughout the study and no implants were removed. No subject required a surgical intervention for a related adverse event.

**Fig. 3:**
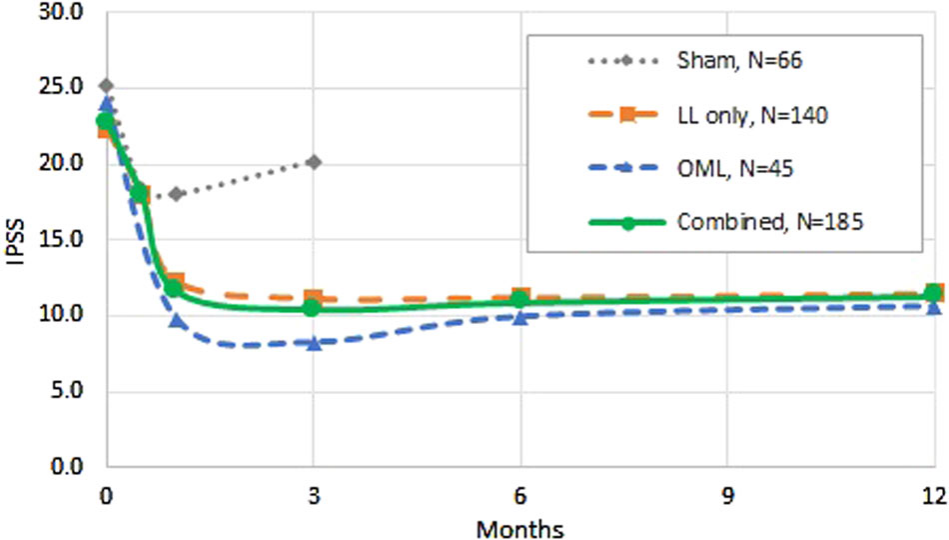
Response to PUL therapy in sham (L.I.F.T. control), LL only (L.I.F.T. active), OML (MedLift), and combined LL with OML (L.I.F.T. active + MedLift) response

**Fig. 4:**
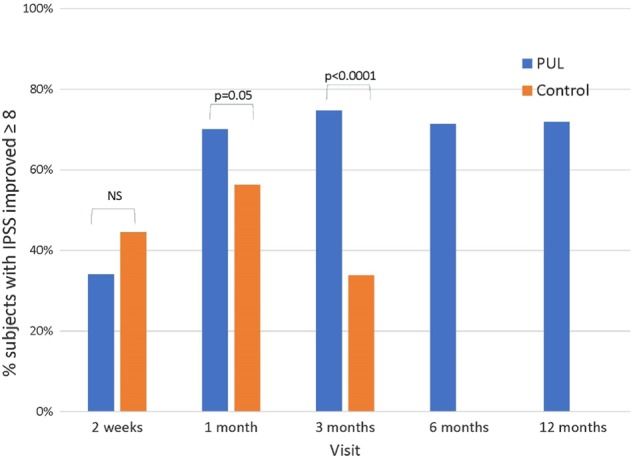
Percent of subjects with IPSS improved 8 points or greater in the combined PUL cohort of LL (L.I.F.T.) and OML (MedLift) study subjects

Symptom improvement for OML subjects was at least as great as that for LL subjects from the L.I.F.T. study at every time point (Fig. 3). When the total FDA IDE study population was integrated (OML with LL data), a strong responder trend was manifested in the combined cohort: 75% of subjects demonstrated an 8 point or greater improvement in IPSS versus 34% in the control group at 3 months (*p* < 0.0001); the outcome was sustained through 6 and 12 month follow-up visits (Fig. 4). At 12 months, the IPSS symptom score improvement of the combined cohort was 11.4 points. Further, an in-depth analysis of sexual function response demonstrated that in the combined cohort of sexually active men, 45% of men (35/78) improved the minimal clinically important difference (MCID) in erectile function as measured by IIEF-EF at 3 months and 40% (31/77) achieved the threshold at 12 months (Table 3).

**Table 3:** Subjects improving the minimal clinically important difference in IIEF-EF score at 3 and 12 months after PUL in combined L.I.F.T. and MedLift cohorts (both LL and OML subjects)

	3 Months			12 Months		
IIEF-EF Baseline Severity	n/N	Increase Mean±SD	Increase Range	n/N	Increase Mean±SD	Increase Range
Severe (1–10)	1/19	8.0	8–8	2/21	17.0 ± 5.7	13–21
Moderate (11–16)	12/23	10.1 ± 1.6	8–13	8/21	8.8 ± 3.2	5–15
Mild (17–25)	22/36	5.8 ± 2.3	2–10	21/35	5.3 ± 2.4	2–10
Total Improved (%)	35/78 (45%)			31/77 (40%)		

*IIEF-EF* International Index of Erectile Function erectile function domain, *MCID* minimal clinically important difference (at least 2 point increase for mild ED, 5 for moderate ED, and 7 for severe ED)

## Discussion

This manuscript summarizes the data from the first and only study to date on PUL for OML subjects and demonstrates that PUL may be safely and effectively used in this population. In the recently revised American Urological Association (AUA) guidelines on the surgical management of LUTS attributed to BPH, PUL was recommended as a standard of care option [4]. The recommendation was made based on a substantial body of evidence that included the L.I.F.T. randomized study. At the time of the guidelines release, however, insufficient data had been published on OML treatment and consequently the recommendations excluded those patients.

It is important to identify the presence of OML since having a mechanical obstruction is related to a higher failure rate of medication. Per current AUA guidelines, physicians should consider assessment with ultrasound and/or cystoscopy during evaluation and preoperative testing to assess for size and shape of the prostate prior to surgical intervention [4]. Such imaging can confirm the presence of OML and help select the best therapeutic approach. As reported in a recent study on middle lobe only, TURP patients who suffer from a significant middle lobe with IPP ≥ 10 mm may benefit substantially from treatments which specifically address this obstruction [22]. The PUL procedure was found to be effective irrespective of prostate size and degree of IPP.

As with any new technique, there is a learning curve that must be considered. For treating OML, PUL requires the physician to pull intravesicular tissue into the prostatic fossa and to affix the tissue laterally. The data collected during this study represent the first cases for all physicians with applying this new PUL technique and captures the possible adverse effects associated with the learning curve. Given the new technique, the study protocol allowed for cases to be completed with general anesthesia per physician preference. With increasing experience, some physicians are currently conducting middle lobe cases in the clinic using local anesthesia only.

The increased amount of manipulation associated with treating the middle lobe and physician comfort with a new technique may be reasons for the increased catheterization rate seen in these subjects compared to the LL subjects treated during the L.I.F.T. study. Compared to other therapies with requirements for bladder irrigation, hospital stay, and catheterization [23], the catheterization rate, duration, and short hospital stay (average < 2.5 h) even in OML patients continues to support PUL as the least invasive therapeutic option [23]. Given that the effectiveness of this therapy in OML patients is comparable to other more invasive treatment options including TURP, PVP, and steam injection [3, 7, 24], the risk to benefit ratio for PUL is attractive for those seeking symptom relief, minimal morbidity, and quality of life improvement.

OML subjects reported substantial IPSS improvement at every follow-up time point that was superior to that seen in the previously published parent L.I.F.T. study. The L.I.F.T. study demonstrated through randomized, controlled five-year results a sustained improvement in symptoms (36% IPSS), quality of life (50% QoL; 52% BPHII), and urinary flow rate (44% Qmax) and an acceptably low surgical retreatment rate of 2–3% per year [13]. With 82% reporting some level of improvement with their urinary symptoms at 5 years, most subjects achieved long term satisfaction with PUL. Further, with 10% of patients in the LIFT study requiring implant removal compared to 0% during this study, issues associated with improper implant placement appear to resolve with experience and training.

Study limitations included non-randomized design, use of historical controls, limited long term follow up, and significant differences between the OML and LL only subjects in terms of age and symptoms at baseline. OML subjects, however, were comparable in all analyzed baseline characteristics to the sham control arm of the LIFT study, which provide calibration for the significant improvements seen in both OML and LL active treatment arms.

In summary, the MedLift study demonstrated that outcomes from PUL treatment of OML are not dissimilar to PUL treatment of LL: rapid, significant, and sustained improvements in IPSS, QoL, and Qmax with a minimally invasive adverse event profile and no new onset, sustained erectile or ejaculatory dysfunction.

### Disclaimer

The views expressed are those of the authors and do not reflect the official policy or position of the US Navy, Department of Defense or the US Government.
